# Musty—Yellow

**Published:** 1884

**Authors:** 


**MUSTY** taste, with pain through chest and back: Bor.[7]
**MUTTON**, tasting like, broth: Iod.[5]
**NARES**, seems to come from posterior: Arn., Mag. c.
**NAUSEOUS**: Coc. c., Cop., Dros.,[12] Nux v., Tarent.
— morning: Dros., Ferr., Puls.
— See Offensive.
**NIGHT**, expectoration at, none during day: Alum., Amm. m., Arn., Calc., *Caust.*, Chin. s.,[1] Coc. c.,[1] Kali c.,[1] Euphr., Led., Lyc.,[1] Phos., Raph., Rhod., Sabad., SEPIA, *Staph.*, Sulph.—4.
— **bloody**: *Ferr.*, Mezer., Sulph.
— **mucus**: Agar., Calc., Cycl., Hepar,[6] Sepia, Sil., Sulph.
— **thick**: *Cycl.*, Lyc.
— **transparent**: Calc. s.
— **whitish**: Sepia.
— **yellow**: Lyc.
**NOON**, **bloody**: Sil.
— much tenacious mucus: Bell.[7]
**OFFENSIVE**, in smell: Alum., Ars., Arn.[7] Asaf., Asar.,[24] Aur., Bell., Bry., *Calc.*, Carbo v., Caust., Cham., China, Con., Copaiba, Cupr.,[2] Digit., Dirc., Dros.,[24] Eupion, Fago, Ferr.,[24] Graph., Guai., Hepar, Hura, Ign.,[2] Kreos., Lip., Led., Lyc., Mag. c.,[12] Mag. m., Merc., *Natr. c.*, Nitr. ac., Nux v., Ph. ac., Puls., Rhus, Sabina, Sac. alb., SANG., Senega, *Sepia*, Sil., Squil.,[2] *Stann.*, Sulph., Thuja.—1 and 5.
— in morning: Cupr., Natr. m.
— — — 10 A. M.: Iod.
— even to patient: *Sang.*
— (nauseous), in taste: Ars., Asaf., Bry., Calc., Canth., China, Cina, Cocc., Dig.,[6] *Dros.*, Iod., *Ipec.*, Kali c., Led., *Merc.*, Natr. m., Nitr. ac., Nux v., Phos., PULS., Sabad., Samb., Selen., Sepia, Squil., *Stann.*, Sulph., Zinc.—5.
— See also PUTRID.
**OLEAGINOUS**, in appearance and consistency: Petrol.
**ONIONS**, tasting like: Asaf., Mag. m., Sulph., Sul. ac.—2.
**OPAQUE**: Aqu. petr., Chin. s.
**ORANGES**, tasting like: Phos.[2]
**PAINFUL**: Ars., Elaps, Merc. c.
— as if from heart: Elaps.
**PALE**: Kali b., Lyceps.
—, after oppression at pit of stomach: *Kali b.*
**PARTICLES**: Aqu. petr.
**PASTY**: Kali b.
**PEACH** kernels, tasting like: Laur.[2]
**PEAS**, tasting like raw: Puls., Zinc.
**PEPPER**, tasting like: Acon., Ars., Mezer., Sabad., Sulph.—2.
**PHOSPHORESCENT**: Phos.
**PIECES**, in: Alum.[5]
—, acrid: Nitr. ac., Rhus.—5.
—, green-gray: Sepia.[5]
**PINKISH**: Carbn. h.
**POMEGRANATE**, tasting like: Phos.[5]
**PROFUSE**. See Copious.
**PURULENT**: Acon., Ailan., All. s., Amm. c., *Anac.*, *Ant. t.*,[7] Arg., Arn.,[7] *Ars.*, Asaf., Aur., Bell., Bry., CALC., *Carbo an.*, Carbo v., Cham., CHINA, Cic., Cimex.,[7] Cina, Cocc., *Cod.*, CON.,[4] Cop.,[12] Cupr., *Dros.*, Dulc., *Ferr.*, Graph., Guai., Hepar, Hyos., Ign., Ipec., Kali b., KALI C., Kali iod., *Kreos.*, Lach., Led., LYC., Mag. c., Mag. m., *Merc.*,[4] *Natr. c.*, Natr. m., *Nitrum*, Nitr. ac., Nux m., Nux v., Œna., PHOS., *Ph. ac.*, *Plumb.*, *Puls.*, *Rhus*, Ruta, Sabina, Samb., Sang.,[7] Secale, SEPIA, SIL., *Stann.*, *Staph.*, Stront., *Sulph.*, Verat., Vic., Zinc., Zinc. s.—1 and 5.

**PURULENT**, mixed with bright blood: Arg. n.[7]
— in forenoon: Œna.
— in morning: Mag. c.,[1] Phos.[6]
— — on waking: Ferr.
— smoking and brandy, agg. by: Ferr.
— first thin, then thick: Lyc.[6]
**PUTRID ODOR**: *Calc.*, Cupr. m., Con., Guai.,[7] SANG.
**PUTRID TASTE**: Acon., All. s., *Arn.*, *Ars.*, Bell., Bov., Bry., Calc., Carbo an., *Carbo v.*, Caust., *Cham.*, Cocc., Con., Cupr., Dros., Ferr., Ham.,[7] Iod., Ipec., Kali c., Kalm.,[6] Kreos., Led., Lyc., Merc., *Natr. c.*, Nux v., Phos., Ph. ac.,[12] *Puls.*, Rhus, Samb., Sepia, Sil., *Stann.*, Staph., Sulph., Verat., Zinc.—1 and 5.
— morning: Cupr., Digit., *Ferr.*—6.
— sweet: Sepia, Zinc.—5.
**RANCID**, taste: Ambr., Bar., Bry., Caust., Cham., Ipec., Lach., Merc., Mur. ac., Nux v., Phos., Puls.—2.
**REDDISH**: Bry.,[12] Coc. c., Dros., Kali b., Kali iod., Œna., Phos.,[12] Squil., Thuja.—1 and 5.
**REPULSIVE TASTE**: Bry., China, Merc., Puls., Sabad., Sepia, Squil., Stann., Zinc.—2.
**RISING** after: Coca.
— See Bed and Morning.
**ROPY**: Coc. c., Kali b., Lobelia.—5.
— See also Stringy.
**RUSTY**: Acon.,[1] Atro.,[1] *Bry.*, Lyc., *Phos.*, Rhus, Sang.,[7] Squilla.—5.
**SALIVA-LIKE**: Cannab. f.
— — in morning: Thuja.
— frothy: Ars.[7]
**SALTY**: Acon., Agar., Alum., Ambra,[7] Amm. c., Ang., Ant. t., Aralia, ARS., Bar., Bell., Bov., *Calc.*, Cann. s., *Carbo v.*, *China*, Cocc., Con., Cop., Dros., Euph., Graph., Hyos., Iod., Kali b.,

**SALTY**—(*Continued*).
Kalm., Lach., Lep., LYC., Mac., Mag. c., Mag. m., Merc., *Merc. sol.*, Merc. c., Mezer., Natr. c., Natr. m., Nitr. ac., *Nux m.*, Nux v., *Phos.*, Plan., *Puls.*, Raph., Rhus, Sac. alb., Samb., *Sepia*, Sil., *Stann.*, Staph., Sulph., Sul. ac., Tarax., Tarent., Therid.,[12] Verat., Wies.—1 and 5.
— with pain in chest while raising; preceded by oppression of chest: Ars.[6]
— when coughing: Ambra.
— as from hawking: Phos.
— morning: Phos.,[6] Ph. ac., Puls.
— sweetish: Mag. m.[6]
**SCANTY**: Acon., Alumen, Apis, Apoc. c., Ars., Brom., Calc. s., Cham.,[12] Clem., Cot., Cupr., Digit., Ery. a., *Ferr.*, Kali b., Lach., Lip., Lyc., Op., Pæon., PHOS., Phyt., *Puls.*, Samb.,[12] Sang.,[12] Sepia, Sil., Spongia,[12] STANN., Tarent.—1 and 5.
— during day: Ailan.
— on going to sleep: Lyc.
— tenacious round lumps, cherry-colored: Acon.[12]
**SEA** bathing, bloody expectoration after: Mag. m.
— weed, tasting like: Spong.[5]
**SELDOM**: Apis.
— See Infrequent.
**SEROUS**: Sulph.
**SIDE**, expectoration is easier when turning from left to right side: Kali c., Lyc., Phos., Sepia, Thuja. —12.
— evening, after lying down, sputum is loose and easier when he turns from left to right side: Thuja.[7]
**SIT UP**, must, at night to raise the sputa: Ferr.
— on sitting up in bed, expectoration: *Phos.*

**SKIN**, like dead: Merc. c.
**SLATE** colored: Kali b., Natr. ars.
**SLIMY**: Acet. ac., Agar., *Ant. t.*, *Arn.*,[7] Asaf., Calc. s., Carbo v., Coc. c., China, Cinch., Cina, Daph., Ipec., Lach.,[7] Lyc., Merc-i-rub., Nux m., *Sabad.*, Spongia.—1.
— **afternoon**: Chin. s.
— — 5.30 P. M.: Hydras.
— **bloody** expectoration: Ant. t.[7]
— during **cough**: Phos.
— when **drinking**: Amm. caus.
— **morning**: Squil., Sul. ac.
— slimy expectoration at times globular and gray, at others viscid and yellow, at others watery; scarcely ever in the night: Lach.[6]
— scanty difficult expectoration of transparent gray slime mixed with black dots or blood: *Arn.*[7]
**SMOKY** taste: Bry., Nux v., Puls., Rhus, Sepia.—2.
**SMOOTH**, easy, gray expectoration, tasting putrid and saltish: Kalm.[7]
**SOAP-LIKE**: Arg. n., Ph. ac.—1.
**SOAPY** taste: Bar., Dulc., Iod., Merc.—2.
**SOFT**, fetid tubercles, color of peas: Mag. c.[12]
**SOUR**, smelling: Calc., Cham., Dulc., Merc., Nitr. ac., Nux v., Sulph., Sul. ac.—2.
—, taste: Ambr., Ang., Ant. t., Ars., Bell., Bry., *Calc.*, Cann. s., Carbo an., Carbo v., Cham., China, Coc. c., Con., Crot. t., Dros., Ferr., Graph., Hepar, Hyos., Ign., Iod.,[7] Ipec., Kali c., Lach., Laur., Lyc., Mag. m., Mag. s., Merc., Natr. c., Natr. m., Nitr. ac., Nitrum, Nux v., Petro., Phos., Ph. ac., Plan., Plumb., Puls., Rhus, Sabin., Sepia, Stann., Sulph., Sul. ac., Tarax., Verat.—1 and 5.
**SOUR**, day: Mag. c.
— night: Hepar.
**STARCH**, like: Agar., Arg.,[1] Bar., Cact. gr.,[1] Dig., Laur., Natr. ars.[1]—5.
— like boiled: Arg., Bar., Cact. gr., Dig.—5.
— — morning, after rising: Coca.
**STICKY**. See Viscid.
**STREAKED**: Pau. p.
— with blood. See under Bloody Expectoration.
**STRETCHING** out between fingers: Chin. s.
**STRINGY**: Æsc. h., Agar., *Arg.*, Arum tr., Asaf.,[2] Coc. c., Ery. a., Ferr., Hydras., Iberis, KALI B., Lach., Lobel., Ruta, Sticta.—1.
— forenoon: Calc. s.
— yellow: Arum tr.[5]
**SUGAR**, tasting like: Calc., Lyc., Sepia.—2.
**SULPHUR**, tasting like: Nux v., Phos., Ph. ac., Sulph.—2.
**SUPPRESSED**: Ant. t., Con., Ipec.
**SWALLOW**, must, what has been loosened: *Arn.*, Calad., Cann. s., *Caust.*, Coca, Con., Dig., Dros., Eugen.,[10] *Kali c.*, Lach., Mur. ac., *Nux m.*, Osm., Sepia, *Spong.*, Staph., Zinc.[1]—5.
— — — at 11 P. M.: Coca.[1]
**SWEETISH**, taste: Acon., Alum., Amm. c., Anac.,[2] Ant. s., Ant. t., Apis,[7] Ars., Asar., Aur., *Calc.*, Canth., China, Coc. c., Cocc., Dig., Ferr., Hepar, Iod., Ipec., Kali b., Kali c., Kreos.,[7] Kobalt.,[7] Laur., Lyceps., Lyc., Merc., Mezer.,[1] Nux v., PHOS., *Plumb.*, *Puls.*, Rhus, *Sabad.*, Samb., Selen., Sepia, Squil., *Stann.*, Sulph., Sul. ac., Zinc.—5.
— — 10 A. M.: Iris v.
— — 2 P. M.: Laur.
— — 5 P. M.: Mag. c.

**SWEETISH**, disagreeable taste: Sepia.[5]
— evening: *Calc.*, Cann. s.
— putrid taste: Sepia, Zinc.—5.
**TAR**, tasting like: Con.[2]
**TASTE**, bad: Lach., Puls.
— — evening: Lach.
**TASTELESS**: Amm. m.,[6] Apis,[7] Arg., Calc.,[6] Cina, *Dulc.*, Par.—5.
— whitish, thick tasteless expectoration in morning: Amm. m.[6]
**TEETH**, smelling and looking like what collects around the teeth when brush is not used; soft like cheese: Aqu. petr.
**TENACIOUS**: Acet. ac., Aloe, Alum., Aqu. petr., Arum it., Aspar., *Carbo v.*, *Coc. c.*, Crot. t., Dig., Dulc.,[6] Hyper., Indigo, *Kali b.*, Lept., *Lac. ac.*, Lach., Lyc., Mag. c., Mag. m., Merc. sul., Mezer., Natr. c., Natr. m., Pæon., Par., *Phos.*, Raph., Ruta,[6] Sang., Secale, Seneg., Sepia, Sul. ac., Thuja, Uva ur., Zinc.[7]—1.
— afternoon: Anac., Ars.
— clear mucus, with painful concussion in the scapulæ and vomiting of bile: China.[6]
— cough, during: Op., Wies.
— morning: Thuja.
**THICK**: Acon.,[16] Aloe, Ambra,[7] Amm. m.,[6] Arg., Ars., Aqu. petr., Arum it., Atrop., Bry., Calc., Carb. ac., Chlo., Cot., Erio., Ery. a., Eupion, Ferr., Feru., Ham.,[7] Hura, Ipec., Kali b., Kobalt,[12] Kreos.,[7] Laur., Lep., Lyc, Merc-i-rub., Naja, Ol. jec., Ox. ac., Phos., Phyto.,[7] Puls., Raph., Ruta, Sang., *Stann.*, Stram.,[1] Sulph., Tarent., Ust., Zinc.—1 and 5.
— afternoon: Eucalyptus.
— evening: Kreos., Sulph.
— **morning**: Agar., Fran., Sulph., Thuja.
— — after getting up: Sulph.

**THICK, morning**, after waking: Lyc.
— **night**: *Cylc.*, Lyc.
— — when cold air: Sac. alb.
**THIN**: All. sat., Bry., Colch., Cupr., Daph., Ferr., Mag. c.,[6] Nitrum.
— viscid, thin phlegm in morning: Ant. cr.[6]
— after supper: Iberis.
— during walk in air: Sac. alb.
— See also Watery.
**THROAT, collection** of mucus in: Acon., Alco., Alumen, Alum., *Ambr.*, Amm. m., Ant. t., Arg., Arn., Ars., Arum d., Asar., Aur., Bar., Bell., Ben. ac., Borax, Bov., Bry., Bufo, Cact. gr., Calc. s., Calo., Carbo an, *Carbo v.*, Caust., Cer. s., Chenop., Cimic., Colch., Croc., Dulc., Ery. a., Eupion, Ferr. iod., Fluor. ac., Graph., Grat., Gymno., Hepar, Hydropho., Hydras., Iod., Indigo, Jug. r., *Kali c.*, Kali iod., Kalm., Kis., Kreos., Lach., Lact., Laur., Lobel., Lyc., Mag. c., Mag. s., Merc., Mer-i-fl., *Mezer.*, Mur. ac., Myric., Natr. ars., Natr. c., Natr. ph., Nit. d. s., Nitr. ac., Nux jug., Ol. an., Op., Ox. ac., Petr., Phell., Phos., Ph. ac., Phys., Plan., Plat., Pod., *Puls.*, *Ran-b.*, Raph., Rhus, Rumex, Sabad., Samb., Sars., Senega, Sepia, Sil., Sol. t. æ., Spig., *Stann.*, Sum., Sul. ac., Tabac., Tarax., Thuja, Tilia, Verat., Wild., Zinc., Zing.—1 and 12.
— — — — **afternoon**: Cer. s., Eucalyptus.
— — — — **evening**: Alum., Ang.,[12] Bry., Calc. ph., Merl.—1.
— — — — — 4 P. M.: Natr. c.
— — — — — 6 P. M.: Physo.
— — — — — 10 P. M.: Trif. p.
— — — — **midnight**: Arum tr.
— — — — **morning**: All. sat., Ambr., Amm. m., Bov., Caust.,

**THROAT**—(*Continued*).
Cimex, Eupion, Hepar, Kali c., Kreos., Lact, Lyc., Marum, *Natr. m.*, Petr., Phos., Plat., Puls., Rhus, Tarax.—1 and 12.
— **collection** of mucus in, **morning**, on waking: Carbo an., Sulph.
— — — — — — — yellow mucus: Apoc. c.
— — — — **night**: Alum., Natr. sul., Puls., Sepia.[1]—12.
— — — — — on waking: Alum.
— expectoration from the, 8.30 A. M.: Naja.
— — —, on getting anything in the: Amm. caus.

**TICKLING**: Caust., Iod.—5.

**TOBACCO** juice, tasting like: Puls.[5]

**TOUGH**: Æsc. h., Acon., Agnus, Ambr.,[7] Anac.,[7] Atropia, Aur., Bov., Bry., Carl., Caust., Coc. c., Iris v., Kali b., Kali c., Kobalt., Merc-i-rub., Phos.,[7] Phyt., Puls. n., Sang.,[7] Senecio, Sil., Tarent., Thuja, Verat.,[7] Vinca.—1.
— after **eating**: Thuja.
— almost causing strangulation and vomiting: Coc. c.[2]
— **morning**: Petro.
— — in bed: Calc.
— — 9 A. M.: Phyt.
— purulent and white: Phos.[7]
— hard to separate, round lumps, brick shade: Bry.[12]

**TRACHEA**, expectoration from the: Carl., Lyc., Marum, Par., STANN.—1.
— **mucus** in the, collection of: Æth., Agnus, Ambr., Amm. c., *Ang.*, Arg., Arn., Ars., Arum d.,[1] *Arum tr.*, Aur., Bar., Bell., Bov., Bry., Cainca,[1] Calc., *Camph.*, Cann. s., Caps., Caust., Cham., China, Cina, Cocc., Coc. c., Croc., Crot. t., Cupr., Digit., Dros., Dulc., Ferr.,

**TRACHEA, mucus** in—(*Cont'd*).
Hyos., Iod., Iris v.,[1] Kali b., Kreos., Lach., Laur., Lyc., Mag. m., Merc. sul.,[1] Natr. m., Nux v., Olean., Osm., Ox. ac., *Par.*,[6] Phell., Phos.,[1] Plumb., Rumex, Samb., Senecio, *Senega*, STANN., Staph., Sulph.—12.
— — — forenoon: STANN.
— — — night: Thuja.
— — — See under Throat.
— — — constant gagging and hawking on account of viscid green mucus in larynx and in trachea: Paris.[7]

**TRASPARENT** (clear, pellucid): Agar., Alum., Ant. t., Apis, Aqu. petr., Arn.,[7] Ars.,[12] Bry., Ferr.,[16] Kali b., Laur.,[12] Phos., Selen.,[6] Senec., Senega,[12] Sil.[16]—1.
— morning: *Selen.*
— night: Calc. s.

**TUBERCLES**: Hepar, Phos.
— brown: Phos.

**URINE**, tasting like: Phos., Senega. —2.

**VIOLETS**, odor of: Phos., Puls.—2.

**VISCID** (gelatinous, gluey): Acon., Agar., Agnus., Ailan.,[1] Alum., Ambr., Am. bro.,[1] Amm. m., Ant. cr., Ant. t., Arg.,[10] Ars., Asar.,[24] Bad., Bar., Bell., Bor., Bov., Bry., Cact. gr., Calc., Calc. s.,[1] *Cann. s.*, Canth., Carbo v., Caust., Cham., China, Cocc., Coc. c., Colch., Cupr., Dulc., Euphr., Ferr.,[24] Graph., Hell., Hepar, Hyper., Iberis, Iod., Kali b., Kali c., Kobalt., Kreos., Lyc., Mag. c., Mag. m., Mezer., Natr. ars., Natr. c., Nux v., *Paris*, Petro., *Phos.*, Ph. ac., Plumb., Puls., Rhus, Ruta, Sabad., Sabin., Samb., Senega, Sepia, Sil., Spig., Spong., Squil., *Stann.*, *Staph.*, Sulph., Tep., Ton., Ust., Verat., Wies., Zinc.—1 and 5.

**VISCID**, afternoon, in the: Naja.
—, morning: Bry.
**WAKING**, after, in morning, thick expectoration: Lyc.
— — — — mucous expectoration: Sulph., Thuja.
— — — — whitish expectoration: Carbo v.
— on: Ferr., Ph. ac.
— — morning, tough yellow sputum: Aurum.[7]
**WALKING**, in open air, agg. Nux v.
— expectoration after: Ferr.[6]
— bloody expectoration while: Cham., Merc., Sul. ac.
— — — — morning: Zinc.
— mucous expectoration while: Natr. m.
**WARM**: Aralia.
**WATER**, tasting like dirty: Acon.[2]
**WATERY**: Acon. l., Agar., Amm. c., Amm. m., Ang.,[1] Aqu. petr., Arg., *Ars.*, Bov., Carbo an., *Carbo v.*, *Cham.*, China, *Daph.*,[6] Euphr., Ferr.,[11] *Graph.*, Guai., Jac.,[1] *Lach.*,[1] Lyc.,[1] *Mag. c.*, Mag. m., *Merc.*, Mezer., Mur. ac., Natr. c., Natr. m.,[1] Nux v., Op., Phos.,[1] Phys.,[1] Plumb., Puls., Ran. sc.,[11] Sac. alb., Sepia, Squil., *Stann.*, Sulph., Sul. ac.—5.
— which does not relieve the cough: Arg.[6]
—, morning: Thuja.[1]
—, mucus: Lach.[5]
**WEATHER**, on change to colder: Lyceps.
**WHITE**, like white of egg: Amm. m., Arn., Ars., Bar., Bor., Bov., China, Coc. c., Ferr., Kali b., Laur., Mezer., Petr., Senega, Sil., Stann.—5.
**WHITISH**: *Acon.*, Ailan., Agar., Ambr., Amm. m., Ant. t., *Arg.*, Arundo, Caps., Carbo an., *Carbo v.*, China,[11] Chin. s., Chlo., Cina,

**WHITISH**—(*Continued*).
Coc. c., Crot. t.,[2] Cupr.,[11] Erio., Ferr., Gad., Hyper., Iberis, Illicium,[7] Iod.,[7] Kali b.,[2] Kali iod., Kobalt., *Kreos.*, Laur., LYC., Manc., Merc-i-rub., Nicc., Œna., Ol. jec., Par., *Phos.*, Ph. ac., Plmbg., *Puls.*,[11] Puls. n., Raph., Rhus, Selen., Senecio, Senega, SEPIA, Sil., Spong., Squil., Stront., *Sulph.*, Tarent., Tellur., Thuja.—1 and 5.
— when in air: Sac. alb., Sepia.
— as from cough: Phys.
— with blackish granules: China.[6]
— like white of an egg: Amm. m., Arn., Ars., Bar., Bor., Bov., China, Coc. c., Ferr., Kali b., Laur., Mezer., Petr., Senega, Sil., Stann. —5.
— evening: Calc. s., Crot. t.
— frothy: Kobalt., Phos., Sil., Sulph.—5.
— gray: Ambra, Sepia.
— morning: Canna.
— — during cough: Sulph.
— — after waking: Carbo v.
— night: Sepia.
— yellow: Lyc., Ox. ac., Ph. ac.—5.
— yellowish white, morning: Lyc.[1]
**WIND**, in cold: Lyceps.
**WINE**, tasting like: Bell., Bry.—2.
**WOOD**, tasting like: Ars., Ign., Stram., Sulph.—2.
**WORKING**, bloody expectoration while: Merc. sol.
**YELLOW**: *Acon.*, Ailan., Aloe,[7] Alum., Ambr., Amm. c., *Amm. m.*, Anac., Ang., Ant. cr., Arg., *Arg. n.*,[7] *Ars.*, Aur., Aur. mur.,[11] Bad., Bar., Bell., Bism., Bor., Bov., Brom., *Bry.*, Cact. gr., CALC., Calc. ph.,[7] Calc. s., Cann. s., Carbo an., *Carbo v.*, Caust., Cham., Chlor.,[11] Cic., Coca, Coc. c., Coloc., Con., Cop., Cupr., Daphn.,[11] Digit., Dign., *Dros.*,

**YELLOW**—(*Continued*).
Eugen., Eupion, Ferr., Feru., Gels., Graph., Ham.,[7] Hepar, Hura, *Ign.*, Iod., Ipec., Kali b., Kali c., *Kreos.*, Lach.,[6] Linu., *Lyc.*, Mag. c., Mag. m., Mang., *Merc.*, Merc-i-r., Mezer., Mur. ac., Natr. ars., *Natr. c.*, Natr. m., *Nitr. ac.*, Nux v., Œna., Op., Ox. ac., Par., Pau. p., Petro., PHOS., Ph. ac., Plumb., Psor., PULS., Rumex, *Ruta*, Sac. alb., Samb.,[7] Sabad., Sabin., Selen., Senega, *Sepia*, SIL., Spig., *Spong.*, STANN., *Staph.*, *Sulph.*, Sul. ac., Tarent., *Thuja*, Verat., *Zinc.*—1 and 5.

**YELLOW**: afternoon: Anac.
— — 12–3 P. M.: Calc. s.
— black: Hydro. ac.
— when in cold air: Sac. alb.
— forenoon: Staph.[6]
— green: Kali b., Mang., Sil., Sulph.—5.
— lemon color: Kali c., Lyc., Phos., Puls.—12.
— morning: Ailan.,[7] *Calc.*, *Calc. ph.*,[7] Fran., Lyc., Mag. c.,[6] Mang., *Ph. ac.*, Sil.
— — 7–10 A. M.: Silicea.
— — on waking, tough yellow sputa: Aurum.[7]
— stringy: Arum. tr.[5]
— white: Lyc., Ox. ac., Phos.—5.

